# GLUT10 is a novel immune regulator involved in lung cancer immune cell infiltration and predicts worse survival when transcriptionally downregulated

**DOI:** 10.1016/j.heliyon.2023.e13836

**Published:** 2023-02-17

**Authors:** Lijuan Jian, Qi Wu, Xinping Min, Bowen Li, Min Zhang, Zhiyong Wu, Xiaoping Hu, Zongli Ren, Zhiwei Wang, Zhipeng Hu

**Affiliations:** Department of Cardiovascular Surgery, Renmin Hospital of Wuhan University, 238 Jiefang Road, Wuhan, China

**Keywords:** GLUT10, Lung cancer, Immune infiltration, Prognosis, Immune molecule, Tumor immunity

## Abstract

**Background:**

Glucose transporter 10 (GLUT10) is encoded by the SLC2A10 gene. Our recent investigations have shown that GLUT10 is not only involved in glucose metabolism but also involved in the body’s immune response to cancer cells. However, the role of GLUT10 in tumor prognosis and in tumor immunity has not been reported.

**Methods:**

We knocked down SLC2A10 and performed transcriptome sequencing to analyse the biological function of GLUT10 and found that GLUT10 may be involved in immune signaling. Then, we studied the expression level of SLC2A10 in cancers by the Oncomine database and Tumor Immune Estimation Resource (TIMER) site. We also evaluated the prognostic potential of SLC2A10 in different cancers using the Kaplan‒Meier plotter database and PrognoScan online software. The correlations between SLC2A10 expression and immune infiltrates were analysed by TIMER. In addition, correlations between SLC2A10 expression and gene marker sets of immune infiltrates were analysed by TIMER and Gene Expression Profiling Interactive Analysis (GEPIA). Immunofluorescence staining of cyclooxygenase-2 (COX-2) and GLUT10 in lung cancer tissue and adjacent tissue was performed to confirm our findings from the database research.

**Results:**

Knocking down SLC2A10 widely activated immune and inflammatory signaling. SLC2A10 was abnormally expressed in several tumors. The expression level of SLC2A10 was closely correlated with cancer prognosis. Low SLC2A10 expression was related to poorer prognosis and increased malignancy of lung cancer. Lung cancer patients with low expression of SLC2A10 have a much shorter median survival time than patients with high expression of SLC2A10. SLC2A10 expression is closely related to the infiltration of different types of immune cells, particularly macrophages. Both database research and lung cancer sample research revealed that GLUT10 might modulate immune cell infiltration via the COX-2 pathway.

**Conclusions:**

By transcriptome experiments, database studies, and human sample studies, we found that GLUT10 is a new immune signaling molecule involved in tumor immunity, especially in the immune cell infiltration of lung adenocarcinoma (LUAD). GLUT10 may modulate the immune cell infiltration of LUAD via the COX-2 pathway.

## 1 Introduction

In recent years, studies on the roles of immune responses and metabolism in tumors have been extensively discussed. The expression of some glucose transporters is downregulated while others are upregulated to meet the energy needs of tumor cells. Of the 14 glucose transport proteins, the roles of glucose transporter 1 (GLUT1) have been well established in a wide range of cancer types. GLUT1 overexpression was shown to promote the proliferation, invasion, and migration of malignant cells [[1], [2], [3]]. However, the role of glucose transport protein expression in tumor immunity, especially GLUT10, remains unknown.

GLUT10 is encoded by the SLC2A10 gene, and mutations in SLC2A10 result in hereditary arterial tortuosity syndrome (ATS). To date, the mechanism of ATS is still poorly understood [4]. Meanwhile, the exact biological function of GLUT10 is also unclear [5].

SLC2A10 is most highly expressed in smooth muscle cells and smooth muscle cell-enriched organs such as the aorta, digestive organs, prostate, and thyroid [6].To explore the biological function of GLUT10, we used several effective interference sequences of SLC2A10 to knock down it in vascular smooth muscle cells (VSMCs). Interestingly, transcriptome experiments revealed broad and significant immune and inflammatory signaling activation after SLC2A10 knockdown. Whether and how GLUT10 plays a role in the immune response has never been reported.

The impact of immune infiltration on tumors has been a particular focus in recent decades. Several established databases, such as SEER and TCGA, with detailed survival and gene expression data are available to investigate the involvement and upstream molecules of immune infiltration in tumors.

In this study, we used the public databases Oncomine, PrognoScan, Kaplan‒Meier Plotter, TIMER and GEPIA and IFC (immunofluorescence) of lung cancer specimens to further investigate the potential prognostic value and immunological roles of SLC2A10 expression in tumors.

## 2 Methods

### 2.1 Vascular smooth muscle cell culture

This animal experiment protocol was approved by the Animal Research Ethics Committee of Renmin Hospital of Wuhan University. Male Sprague‒Dawley rats weighing 150–180 g were purchased from the Hubei Province Center for Disease Control and Prevention (China). Euthanasia of study animals was performed by removal of vital organs under deep anaesthesia as stated in the euthanasia policy of the UCSF laboratory (POLICY - Euthanasia.pdf (ucsf.edu)). The experiment was carried out according to the following process: Rats were intraperitoneally anaesthetized with 2% pentobarbital sodium (100 mg/kg, Sinopharm Chemical, Shanghai, PRC), and the aorta was isolated in a sterile environment. The aorta was cleaned of all connective tissue, cut into small pieces and placed in a 6 cm cell culture dish. Then the cells were inoculated with fresh culture medium (DMEM/F12, 20% fetal bovine serum, 1% penicillin/streptomycin) (all from Thermo Fisher, MA, USA). After culturing in a 37 °C incubator with 5% CO2 for 30 min, the medium was replaced after the cells grew and then replaced every 2–3 days. After the VSMCs were fully spread in the culture flask, the original medium was removed, the cells were washed twice with PBS (Servicebio, Hubei, China), 1 mL of 0.5% pancreatin (both from Gibco) was added, the cells were digested at 37 °C for 2–3 min, and complete medium was added to terminate digestion. Finally, VSMCs were inoculated in culture plates. Cells from passage 3 were used in the present study [7].

### 2.2 Gene interference and transcriptome sequencing

Short hairpin RNA (shRNA) was used to knock down SLC2A10 in VSMCs. The effectiveness of gene interference was tested by qPCR. Transcriptome sequencing was performed, and FASTQ files from RNA-seq experiments were clipped and trimmed. KEGG enrichment analysis of differentially expressed genes was performed. The rich factor was calculated as the ratio of the number of genes in the pathway entry in the differentially expressed gene to the total number of genes in the pathway entry in all the annotated genes.

### 2.3 Oncomine database analysis

Oncomine is a database of tumor gene expression that can be used to compare the transcriptome differences between most cancers and their adjacent normal tissues [8,9]. Therefore, we analysed the expression level of SLC2A10 in various types of cancers using the Oncomine database [8]. The threshold was set as follows: P value of 0.001, fold change of 1.5, and gene ranking of all.

### 2.4 PrognoScan database analysis

The Pronoscan database is a cancer microarray dataset with clinical annotations. It is an online platform for evaluating the biological relationship between gene expression and tumor prognosis. To test the correlation between the SLC2A10 expression level and the survival of patients with a series of cancers, we ran an online analysis on the Pronoscan website (http://dna00.bio.kyutech.ac.jp/PrognoScan/index.html) [10]. The threshold was set as a Cox P value < 0.05.

### 2.5 Kaplan‒Meier Plotter database analysis

Kaplan‒Meier plotter was mainly used to evaluate the relationship between gene expression level and tumor prognosis. The primary purpose of the tool is a meta-analysis-based discovery and validation of survival biomarkers. The correlation between SLC2A10 expression and survival in gastric cancer, ovarian cancer, bladder cancer, breast cancer, esophageal adenocarcinoma, kidney renal clear cell carcinoma, liver hepatocellular carcinoma, lung cancer, pancreatic ductal adenocarcinoma, stomach adenocarcinoma and uterine corpus endometrial carcinoma was analysed by Kaplan‒Meier plotter (http://kmplot.com/analysis/index.php?p=service) [11]. Patients were split by median. The follow-up threshold was set as all.

### 2.6 TIMER database analysis

The TIMER database is a strong web tool for evaluating immune infiltration by comparing the expression levels of immune-related genes in tumor tissues. (https://cistrome.shinyapps.io/timer/) [12]. The abundance of immune infiltrating cells was estimated by the expression level of gene markers of T cells (CD8(+), general), B cells, monocytes, macrophages (tumor associated macrophages, M1/M2 phenotype), neutrophils, NK cells, dendritic cells, T helper cells, Tregs, and exhausted T cells using common markers as reported [[13], [14], [15]]. The correlation between SLC2A10 expression and the immune cell infiltration abundance in given cancer types was plotted by the correlation module together with Spearman’s correlation and estimated statistical significance.

### 2.7 Gene correlation analysis in GEPIA

GEPIA is an interactive platform for online analysis of RNA sequencing data in the TCGA and GTEx databases [16]. The correlation between SLC2A10 expression and the markers of immune cells in LUAD and lung squamous cell carcinoma (LUSC) was estimated using GEPIA.

### 2.8 Immunofluorescence staining

Immunofluorescence staining was performed to compare COX-2 and GLUT10 expression in LUAD and adjacent noncancerous tissues. The tissue microarray was purchased from Wuhan Seville company, and the present study was approved by the Ethics Committees of Renmin Hospital of Wuhan University (China). Informed consent was obtained from all subjects and all procedures involving human samples strictly abided by the Declaration of Helsinki. The process of immunofluorescence staining was as follows: routine dewaxing and hydration of the tissue microarray, microwave antigen retrieval, blocking with 3 mg/mL bovine serum albumin, incubation with the primary antibody at 4 °C overnight followed by incubation with the secondary antibody for 1 h, staining with DAPI, and observation under a fluorescence microscope [17]. The primary antibodies used in this study were as follows: anti-COX-2 (Beyotime, AF1924) and anti-GLUT10 (Santa Cruz Biotechnology, sc-398495). All images were quantified using the open-source software Fiji (https://fiji.sc). Five visual fields were randomly selected from each sample, and the fluorescence intensity was calculated.

### 2.9 Immunofluorescence colocalization analysis

To assess the correlation between GLUT10 and COX-2, double IFC staining was used to detect the colocalization of GLUT10 and COX-2 in samples. We used colocalization finder for immunofluorescence colocalization to evaluate the cancer samples and adjacent tissues.

### 2.10 Statistical analysis

We used Student’s *t*-test to compare the differences between the two groups of measurements, while survival curves were generated by PrognoScan and Kaplan‒Meier plots. GraphPad Prism 7.0 software (GraphPad Software, California, USA) was used for statistical analyses. The correlation between the variables was assessed by Spearman’s correlation analysis and Pearson’s correlation analysis, while the credibility of colocalization was assessed by the Manders overlap coefficient test. P < 0.05 was considered to indicate a statistically significant difference.

## 3 Results

### 3.1 Knockdown of SLC2A10 widely activated immune and inflammatory signaling

**Fig. 1:**
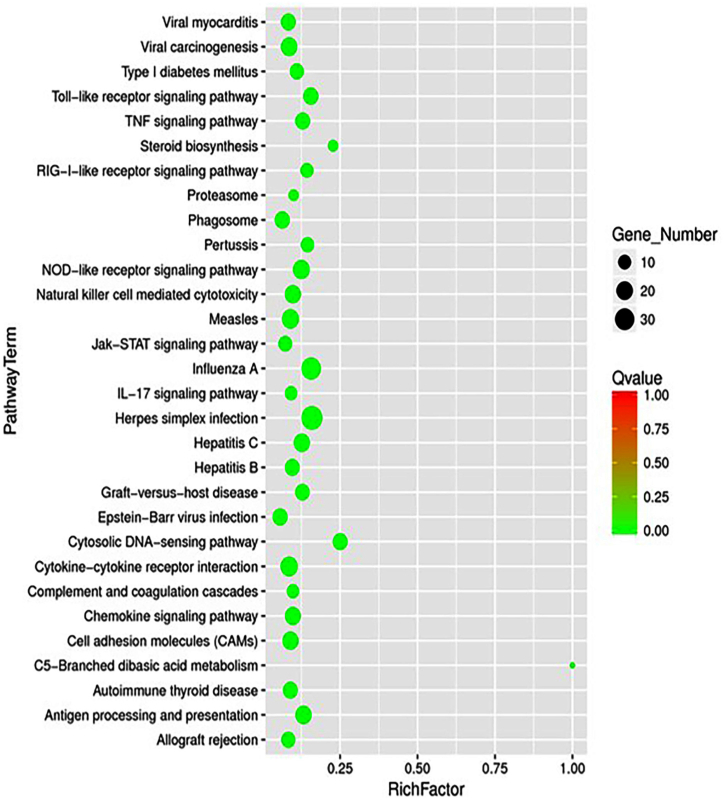
KEGG enrichment analyses of SLC2A10 knockdown.

SLC2A10 is highly expressed in smooth muscle cells. To investigate its biological function, we knocked it down in VSMCs by shRNA with different interference sequences. Transcriptome sequencing was performed in cells infected by shRNA virus or control virus. KEGG analysis revealed wide activation of immune-related pathways, such as the Toll-like receptor signaling pathway, TNF signaling pathway, RIG-I-like receptor signaling pathway, NOD-like receptor signaling pathway, natural killer cell-mediated cytotoxicity pathway, IL-17 signaling pathway, and C5-branched dibasic acid metabolism pathway and linked to some immune-related diseases, such as viral myocarditis, Epstein‒Barr virus infection, hepatitis C, influenza, autoimmune thyroid disease and allograft rejection (Fig. 1). These results suggest a strong immune modulation function of SLC2A10.

### 3.2 SLC2A10 is abnormally expressed in several tumors

**Fig. 2:**
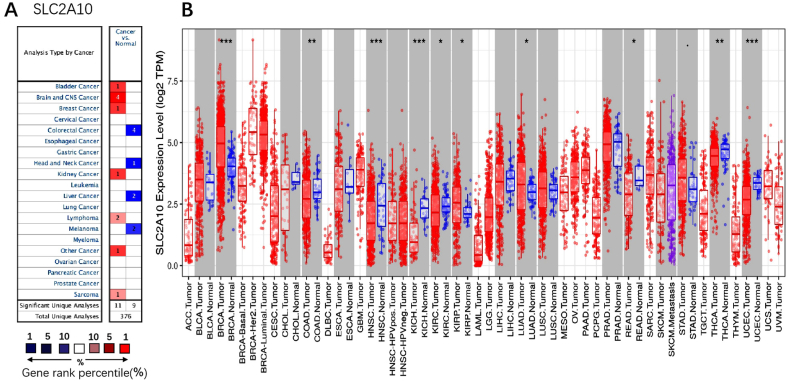
SLC2A10 expression levels in different types of human cancers. (A) Increased or decreased SLC2A10 in data sets of different cancers compared with normal tissue in the Oncomine database. (B) Human SLC2A10 expression levels in different tumor types from the TCGA database were determined by TIMER (*P < 0.05, **P < 0.01, ***P < 0.001).

To investigate the expression level of SLC2A10 in different tumors, the mRNA expression of SLC2A10 in multiple cancer types and their corresponding normal tissues was compared using the Oncomine database. SLC2A10 was expressed at lower levels than in normal tissue in colorectal cancer, head and neck cancer, liver cancer, and melanoma but expressed at higher levels than in normal tissue in bladder cancer, brain and central nervous system cancer, breast cancer, kidney cancer, lymphoma and sarcoma (Fig. 2A).

Then, we used an online tool named TIMER to further confirm the expression level of SLC2A10 in tumors. The differential expression of SLC2A10 between tumor and adjacent normal tissues across all TCGA tumors was compared. SLC2A10 is expressed at low levels in colon adenocarcinoma, head and neck squamous cell carcinoma, kidney chromophobe, thyroid carcinoma, and uterine corpus endometrial carcinoma but is highly expressed in bladder cancer (Fig. 2B). The SLC2A10 expression levels in tumors from the Oncomine database and TCGA database were similar.

### 3.3 Prognostic potential of SLC2A10 expression in tumors

To evaluate whether SLC2A10 impacts the prognosis of cancer patients, we analysed the correlation between the SLC2A10 expression level and patient survival using PrognoScan online software. As presented in Table 1, the SLC2A10 expression level was significantly correlated with worse survival in several cancers, including brain cancer, breast cancer, lung cancer, colorectal cancer, eye cancer, skin cancer, and soft tissue cancer, while it was slightly correlated with worse survival in ovarian cancer.

**Table 1:** Correlation analysis between SLC2A10 expression level and patient survival in PrognoScan.

Cancer type	Survival type	N	Corrected P value
Brain cancer(astrocytoma)	OS	70	0.0206463
Brain cancer(glioblastoma)	OS	74	0.0156356
Brain cancer(glioma)	OS	67	0.00165497
Brain cancer(meningioma)	OS	200	0.0946148
Breast cancer	DMFS	155	0.0505604
Breast cancer	OS	145	0.00571245
Colorectal cancer	DFS	177	0.0431384
Colorectal cancer	DSS	226	0.0916554
Colorectal cancer	DFS	63	0.0836614
Eye cancer(uveal melanoma)	DMFS	82	9.17763E-05
Lung cancer(adenocarcinoma)	OS	204	0.099865
Lung cancer(adenocarcinoma)	RFS	278	0.0273683
Ovarian cancer	OS	110	0.0610717
Ovarian cancer	OS	110	0.093969
Ovarian cancer	PFS	38	0.074999
Skin cancer(melanoma)	OS	140	0.013884
Soft tissue cancer(liposarcoma)	DRFS	77	0.0148117

OS: Overall Survival; DMFS: Distant Metastasis Free Survival; DFS: Disease Free Survival; DSS:Disease Specific Survival; RFS: Relapse Free Survival; PFS: Progression Free Survival; DRFS: Distant Recurrence Free Survival.

**Fig. 3:**
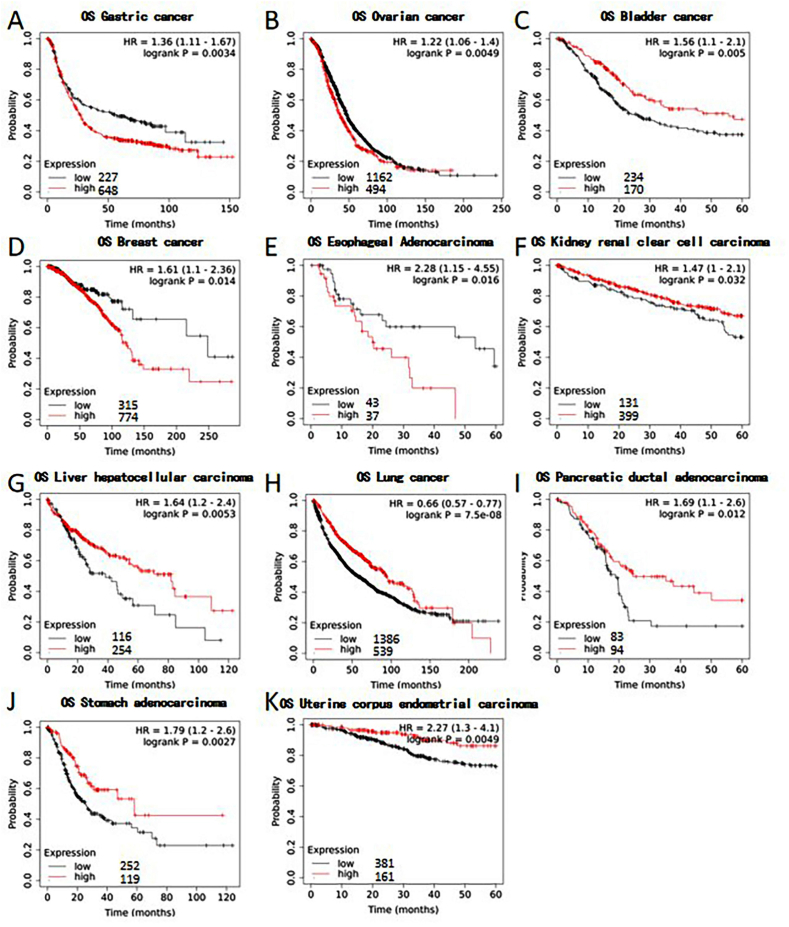
Kaplan‒Meier survival curves comparing the high and low expression of SLC2A10 in different types of cancer in the Kaplan‒Meier plotter databases (A–K). (A) OS survival curves of gastric cancer. (B) OS survival curves of ovarian cancer. (C) OS survival curves of bladder cancer. (D) OS survival curves of breast cancer. (E) OS survival curves of esophageal adenocarcinoma. (F) OS survival curves of kidney renal clear cell carcinoma. (G) OS survival curves of liver hepatocellular carcinoma. (H) OS survival curves of lung cancer. (I) OS survival curves of pancreatic ductal adenocarcinoma. (J) OS survival curves of stomach adenocarcinoma. (K) OS survival curves of uterine corpus endometrial carcinoma. OS, overall survival.

To further examine the prognostic potential of SLC2A10 in different cancers, the Kaplan‒Meier plotter database was used to evaluate the SLC2A10 prognostic value based on Affymetrix microarrays or PCR (only for lung cancer). The expression level of SLC2A10 was significantly correlated with worse survival of patients with gastric cancer, ovarian cancer, bladder cancer, breast cancer, esophageal adenocarcinoma, kidney renal clear cell carcinoma, liver hepatocellular carcinoma, lung cancer, pancreatic ductal adenocarcinoma, stomach adenocarcinoma and uterine corpus endometrial carcinoma (Fig. 3A–K).

### 3.4 Downregulation of SLC2A10 strongly decreases the survival of patients with lung cancer

**Fig. 4:**
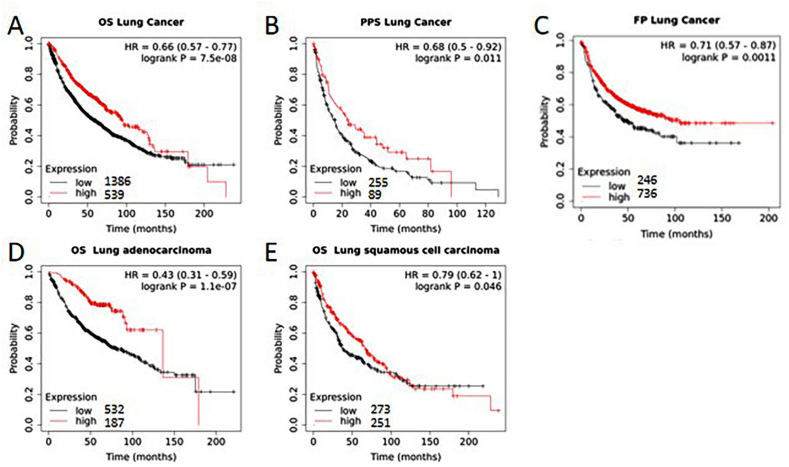
Kaplan‒Meier survival curves comparing the high and low expression of SLC2A10 in histological types of lung cancer in the Kaplan‒Meier plotter databases (A–E). (A) OS survival curves of lung cancer. (B) PPS survival curves of lung cancer. (C) FP survival curves of lung cancer. (D) OS survival curves of lung adenocarcinoma. (E) OS survival curves of lung squamous cell carcinoma.

Among al*l* cancer types, the survival of lung cancer patients is most strongly affected by SLC2A10 expression. Regardless of the type of survival (OS, PPS, FP), lower expression of SLC2A10 predicts the worst prognosis of lung cancer. Lower expression of SLC2A10 was also correlated with shorter median survival months. When histological type was considered, adenocarcinoma was more significantly correlated with worse survival (p < 0.0001) than squamous cell carcinoma (p = 0.046) (Fig. 4A–E). Patients with low expression of SLC2A10 in lung cancer had a much shorter median survival time than patients with high expression of SLC2A10 (Table 2).

**Table 2:** The survival time of lung cancer patients with low and high expression of SLC2A10.

Histology	Survival type	Low slc2a10 (months)	High slc2a10 (months)
Lung cancer	OS	59	95
Squamous cell carcinoma	OS	37.63	67
Adenocarcinoma	OS	79.87	136.33
Lung cancer	PPS	15	24.4
Lung cancer	FP	50	104.9

OS: Overall Survival; PPS: Post Progression Survival; FP: First Progression.

### 3.5 SLC2A10 expression correlates with immune cell infiltration in several tumors including lung cancer

**Fig. 5:**
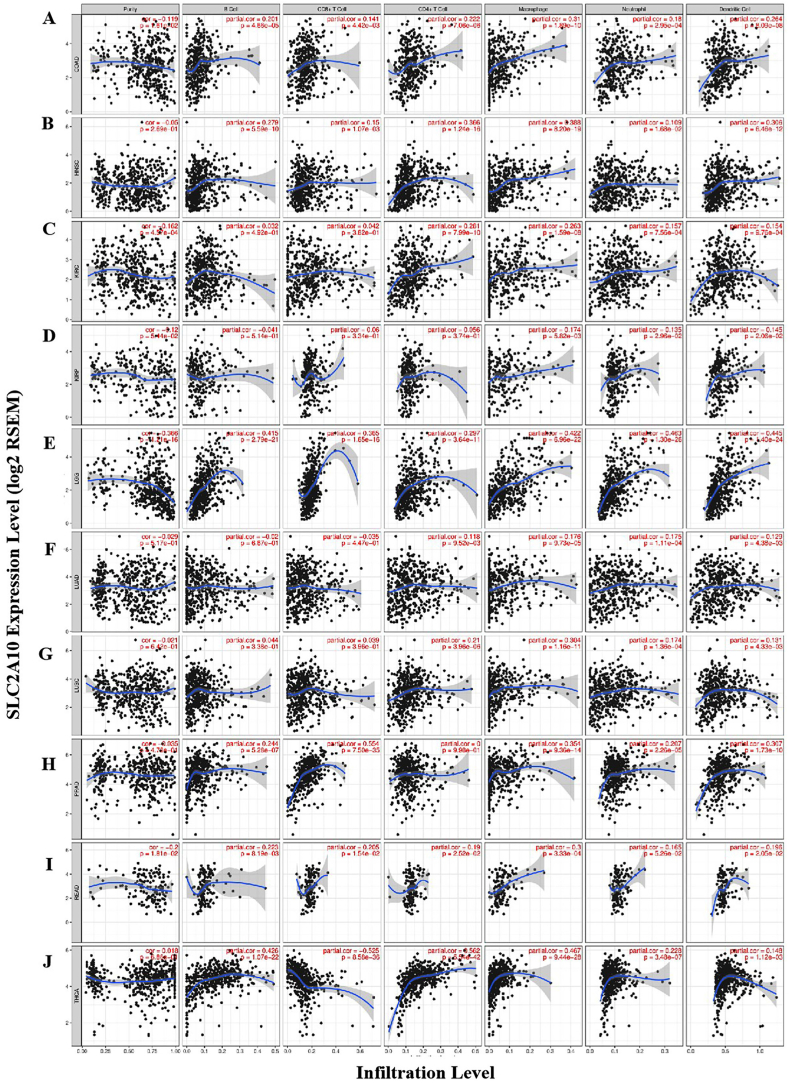
Correlation of SLC2A10 expression with immune infiltration level in COAD (colon adenocarcinoma), HNSC (head and neck cancer), KIRC (kidney renal clear cell carcinoma), KIRP (kidney renal papillary cell carcinoma), LGG (lower grade glioma), LUAD (lung adenocarcinoma), LUSC (lung squamous cell carcinoma), PRAD (prostate adenocarcinoma), READ (rectum adenocarcinoma) and THCA (thyroid carcinoma). (A) SCL2A10 expression levels have a significant positive correlation with infiltrating levels of B cells (B) SCL2A10 expression levels have a significant positive correlation with infiltrating levels of B cells, macrophages, neutrophils, and dendritic cells in HNSC. (C) SCL2A10 expression levels were significantly positively correlated with infiltrating levels of CD4^+^ T cells, macrophages, neutrophils, and dendritic cells in KIRC. (D) SCL2A10 expression levels were significantly positively correlated with infiltrating levels of Macrophages, Neutrophils, and Dendritic cells in KIRP. (E) SCL2A10 expression levels were significantly positively correlated with infiltrating levels of B cells, CD8^+^ T cells, CD4^+^ T cells, macrophages, neutrophils, and dendritic cells in LGG. (F) SCL2A10 expression levels were significantly positively correlated with infiltrating levels of CD4^+^ T cells, macrophages, neutrophils, and dendritic cells in LUAD. (G) SCL2A10 expression levels were significantly positively correlated with infiltrating levels of CD4^+^ T cells, macrophages, neutrophils, and dendritic cells in LUSC. (H) SCL2A10 expression levels were significantly positively correlated with infiltrating levels of B cells, CD8^+^ T cells, macrophages, neutrophils, and dendritic cells in PRAD. (I) SCL2A10 expression levels were significantly positively correlated with infiltrating levels of B cells, CD8^+^ T cells, CD4^+^ T cells, macrophages, neutrophils, and dendritic cells in READ. (J) SCL2A10 expression levels were significantly positively correlated with infiltrating levels of B cells, CD4^+^ T cells, macrophages, neutrophils, and dendritic cells in READ.

Some research shows that tumor-infiltrating lymphocytes are an independent predictor of sentinel lymph node status and an effective marker for evaluating prognosis in some cancers [18,19]. Therefore, we used the gene module of TIMER to explore the correlation between gene expression and the abundance of immune infiltrates in different types of cancers. We found that SLC2A10 expression had significant correlations with tumor purity in 5 types of cancers and significant correlations with macrophage infiltration levels in 22 types of cancers. Furthermore, the expression of SLC2A10 was significantly correlated with the infiltration levels of DCs in 19 types of cancers, neutrophils in 16 types of cancers, CD8^+^ T cells in 13 types of cancers, and CD4^+^ T cells and B cells in 11 types of cancers. In these results, we further found that SLC2A10 expression was significantly associated with the level of immune infiltrates in 10 types of these cancers. As the figure shows, the SLC2A10 expression level correlates with poorer prognosis and high immune infiltrates in colon adenocarcinoma (COAD), kidney renal clear cell carcinoma (KIRC), lower grade glioma (LGG) and rectum adenocarcinoma (READ) (Fig. 5A, C, E, I). In addition, SLC2A10 expression had no significant correlation with tumor purity but had a significant positive correlation with infiltration levels in head and neck cancer (HNSC), kidney renal papillary cell carcinoma (KIRP), LUAD, LUSC, prostate adenocarcinoma (PRAD) and thyroid carcinoma (THCA) (Fig. 5B, D, F, G, H, J). These findings strongly suggest that SLC2A10 plays a specific role in immune infiltration in some cancers, particularly in macrophages.

### 3.6 Correlation analysis between SLC2A10 expression and immune marker sets

To further investigate the relationship between SLC2A10 and diverse immune infiltrating cells, we used the correlation analysis module of TIMER and GEPIA to measure the correlations between SLC2A10 and immune marker sets of various immune cells of LUAD and LUSC. We analysed the correlations between SLC2A10 expression and immune marker genes of different immune cells, including CD8^+^ T cells, T cells (general), B cells, monocytes, TAMs, M1 and M2 macrophages, neutrophils, NK cells, DCs, Th1 cells, Th2 cells, Tfh cells, Th17 cells, Tregs and exhausted T cells in LUAD and LUSC (Table 3). Tumor purity could lead to systematic bias in the results of genome-based tumor studies. To control this bias, we used correlation adjusted by tumor purity in the correlation analysis module of TIMER for correction. The results revealed that the SLC2A10 expression level was significantly correlated with most immune marker sets of various immune cells in LUAD and LUSC. Interestingly, we found that the expression levels of most marker sets of M1 macrophages, monocytes, TAMs, neutrophils, DCs, Th1 cells, Th2 cells and Tregs had strong correlations with SLC2A10 expression in LUAD and LUSC (Table 3). Specifically, we found that COX-2 (PTGS2) in M1 macrophages and GATA3 in Th2 cells were significantly correlated with SLC2A10 expression in LUAD and LUSC. In addition, we found that IRF5 in M1 macrophages and CCL2 in TAMs were significantly correlated with SLC2A10 expression in LUAD. We showed that INOS (NOS2) of M1 macrophages, CD11b (ITGAM) of neutrophils, BDCA-4 (NRP1) of DCs, STAT6 of Th2 cells, STAT3 of Th17 cells and TGFβ (TGFB1) of Tregs were significantly correlated with SLC2A10 expression in LUAD. We further analysed the correlation between SLC2A10 expression and the above markers of M1 and M2 macrophages, TAMs and Th2 cells in the GEPIA database, including LUAD and LUSC. The correlation between SLC2A10 and TAM markers was in accordance with TIMER research (Table 4). These findings suggest that SLC2A10 may regulate macrophage polarization in LUAD and LUSC. Therefore, these results further confirm the findings that SLC2A10 is specifically correlated with immune infiltrating cells in LUAD and LUSC, which suggests that GLUT10 plays a vital role as a protective antigen in lung cancer.

**Table 3:** Correlation analysis between SLC2A10 expression level and related genes and markers of immune cells in TIMER.

Description	Gene markers	LUAD				LUSC			
		None		purity		None		Purity	
		Cor	P	Cor	P	Cor	P	Cor	P
CD8^+^ T cell	CD8A	−0.073	0.0985	−0.107	0.0179	−0.004	0.929	−0.021	0.649
	CD8B	−0.057	0.195	−0.078	0.0822	0.081	0.0697	0.066	0.153
T cell (general)	CD3D	−0.061	0.169	−0.095	0.0342	−0.04	0.368	−0.066	0.15
	CD3E	−0.042	0.339	−0.077	0.0893	0.017	0.696	−0.001	0.985
	CD2	−0.04	0.361	−0.068	0.132	−0.012	0.792	−0.032	0.0481
B cell	CD19	0.011	0.808	−0.013	0.772	0.049	0.274	0.034	0.475
	CD79A	−0.005	0.913	−0.032	0.473	0.064	0.152	0.053	0.252
Monocyte	CD86	0.072	0.105	0.069	0.128	0.098	0.0281	0.106	0.0206
	CD115(CSF1R)	0.124	*	0.126	*	0.12	*	0.132	*
TAM	CCL2	0.201	***	0.208	***	0.1	0.0247	0.107	0.0199
	CD68	0.134	*	0.134	*	0.038	0.402	0.037	0.416
	IL10	0.061	0.164	0.061	0.176	0.109	0.0147	0.115	0.012
M1 Macrophage	INOS(NOS2)	0.052	0.24	0.056	0.241	0.24	***	0.238	***
	IRF5	0.15	**	0.154	**	0.058	0.192	0.068	0.138
	COX2(PTGS2)	0.222	***	0.208	***	0.276	***	0.275	***
M2 Macrophage	CD163	0.087	0.049	0.084	0.0625	0.138	*	0.144	*
	VSIG4	0.057	0.199	0.061	0.174	0.089	0.0471	0.092	0.0439
	MS4A4A	0.026	0.561	0.027	0.548	0.082	0.0656	0.085	0.0627
Neutrophils	CD66b(CEACAM8)	0.025	0.579	0.029	0.517	0.12	*	0.107	0.0189
	CD11b(ITGAM)	0.133	*	0.136	*	0.154	**	0.17	**
	CCR7	0.008	0.85	−0.011	0.801	0.087	0.0529	0.079	0.0856
Natural Killer cell	KIR2DL1	−0.131	*	−0.148	*	−0.028	0.535	−0.047	0.308
	KIR2DL3	−0.083	0.0613	−0.081	0.0718	0.054	0.227	0.049	0.285
	KIR2DL4	−0.014	0.743	−0.033	0.459	−0.056	0.209	−0.071	0.119
	KIR3DL1	−0.127	*	−0.132	*	0.031	0.485	0.019	0.686
	KIR3DL2	−0.038	0.387	−0.053	0.236	−0.066	0.142	−0.082	0.0748
	KIR3DL3	−0.041	0.348	−0.036	0.431	−0.068	0.127	−0.075	0.104
	KIR2DS4	−0.061	0.166	−0.066	0.142	0.041	0.363	0.033	0.466
Dendritic cell	HLA-DPB1	0.03	0.492	0.018	0.695	0.045	0.32	0.035	0.447
	HLA-DQB1	0.008	0.857	0.005	0.92	0.023	0.605	0.012	0.786
	HLA-DRA	0.025	0.578	0.014	0.754	0.029	0.518	0.021	0.652
	HLA-DPA1	0.056	0.203	0.047	0.295	0.053	0.24	0.045	0.326
	BDCA-1(CD1C)	0.055	0.215	0.058	0.202	0.059	0.187	0.059	0.198
	BDCA-4(NRP1)	0.034	0.44	0.04	0.381	0.308	***	0.327	***
	CD11c(ITGAX)	0.125	*	0.121	*	0.139	*	0.161	**
Th1	T-bet(TBX21)	−0.075	0.0877	−0.102	0.0231	−0.006	0.901	−0.021	0.654
	STAT4	0.094	0.0324	0.083	0.0665	0.156	**	0.172	*
	STAT1	0.066	0.133	0.055	0.223	0.025	0.572	0.014	0.756
	IFN-γ(IFNG)	−0.123	*	−0.145	*	−0.112	0.0122	−0.129	*
	TNF-α(TNF)	0.12	*	0.136	*	0.053	0.234	0.05	0.275
Th2	GATA3	0.231	***	0.235	***	0.265	***	0.253	***
	STAT6	0.044	0.322	0.048	0.287	0.164	**	0.161	**
	STAT5A	0.139	*	0.139	*	0.141	*	0.148	*
	IL13	0.006	0.899	0.006	0.899	−0.09	0.0444	−0.1	0.0289
Tfh	BCL6	0.103	0..0199	0.092	0.0417	0.126	*	0.125	*
	IL21	−0.081	0.0655	−0.095	0.0348	−0.032	0.48	−0.029	0.534
Th17	STAT3	0.087	0.0487	0.08	0.0768	0.231	***	0.233	***
	IL17A	−0.06	0.175	−0.056	0.217	−0.106	0.0171	−0.112	0.0147
Treg	FOXP3	0.059	0.18	0.053	0.24	0.091	0.0428	0.09	0.0486
	CCR8	0.037	0.405	0.029	0.522	0.105	0.0187	0.104	0.0234
	STAT5B	0.054	0.217	0.052	0.247	0.113	0.0113	0.131	*
	TGFβ(TGFB1)	0.142	*	0.132	*	0.199	***	0.201	***
T cell exhaustion	PD-1(PDCD1)	0.014	0.751	−0.014	0.756	0.022	0.624	0.011	0.819
	CTLA4	−0.056	0.203	−0.085	0.0598	0.008	0.867	−0.004	0.926
	LAG3	−0.023	0.609	−0.047	0.297	−0.051	0.257	−0.069	0.132
	TIM-3(HAVCR2)	0.06	0.177	0.057	0.207	0.028	0.537	0.021	0.649
	GZMB	−0.08	0.069	−0.113	0.0122	−0.093	0.0374	−0.12	*

LUAD, lung adenocarcinoma; LUSC, lung squamous cell carcinoma; TAM,tumor-associated macrophage; Th, T helper cell; Tfh, follicular helper T cell; Treg, regulatory T cell; Cor,R value of Spearman’s correlation; None, correlation without adjustment. Purity, correlation adjusted by purity. *P < 0.01; **P < 0.001; ***P < 0.0001.

**Table 4:** Correlation analysis between SLC2A10 expression level and related genes and markers of macrophages and Th2 cells in GEPIA.

Description	Gene markers	LUAD				LUSC			
		Tumor		Normal		Tumor		Normal	
		R	P	R	P	R	P	R	P
TAM	CCL2	0.18	***	−0.048	0.72	0.055	0.22	−0.19	0.19
	CD68	0.12	0.011	−0.27	0.038	−0.0021	0.96	0.17	0.23
	IL10	0.054	0.24	−0.23	0.077	0.073	0.11	−0.013	0.93
M1 Macrophage	INOS(NOS2)	0.076	0.096	0.49	***	0.25	***	0.42	*
	IRF5	0.14	*	−0.17	0.2	0.05	0.27	0.15	0.28
	COX2(PTGS2)	0.21	***	0.059	0.66	0.24	***	−0.099	0.49
M2 Macrophage	CD163	0.045	0.32	−0.41	*	0.031	0.49	0.28	0.047
	VSIG4	0.047	0.3	−0.34	*	0.025	0.58	−0.057	0.69
	MS4A4A	0.00046	0.99	−0.41	*	0.015	0.74	0.15	0.3
Th2	GATA3	0.22	***	0.43	**	0.23	***	0.47	**

LUAD, lung adenocarcinoma; LUSC, lung squamous cell carcinoma; TAM,tumor-associated macrophage; Th2, T helper cell 2. Tumor, correlation analysis in tumor tissue of TCGA. Normal, correlation analysis in tumor tissue of TCGA. *P < 0.01; **P < 0.001; ***P < 0.000.

### 3.7 Confirmation of the correlation between COX-2 and GLUT10 expression in LUAD

**Fig. 6:**
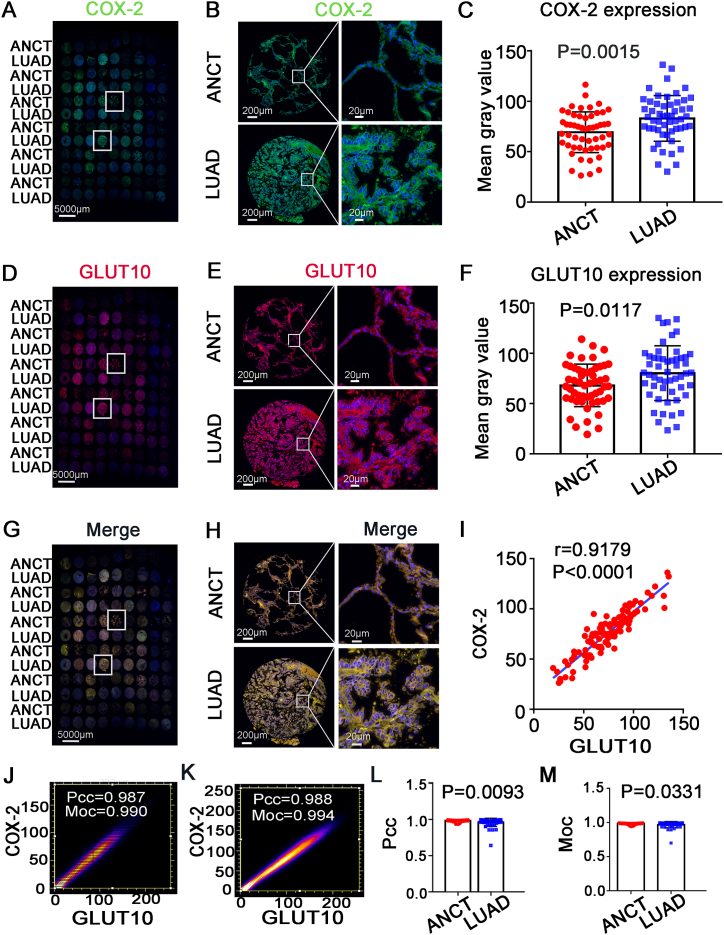
Double immunofluorescence staining tissue microarray. (A) Tissue microarray stained by COX-2 (Green) immunofluorescence. (B) Single tissue stained by COX-2 (Green) immunofluorescence. (C) COX- 2 expression in ANCT and LUAD. (D) Tissue microarray stained by GLUT10 immunofluorescence. (E) Single tissue stained by GLUT10 (Red) immunofluorescence. (F) GLUT10 expression in ANCT and LUAD. (G)–(H) Tissue microarray stained by COX-2 (Green) and GLUT10 (red) immunofluorescence. (I) The correlation coefficient of COX-2 and GLUT10. (J)–(K) Colocalization of COX-2 and GLUT10. LUAD, lung adenocarcinoma tissue; ANTC, adjacent noncancerous tissue in tissue microarray. Pcc, Pearson’s correlation coefficient; Moc, Manders overlap coefficient. (For interpretation of the references to color in this figure legend, the reader is referred to the Web version of this article.)

We further confirmed the involvement of the COX-2 pathway in lung cancer immune infiltration downstream of GLUT10. Tissue microarray immunofluorescence staining showed that the expression of COX-2 and GLUT10 in lung adenocarcinoma tissue was significantly higher than that in adjacent noncancerous tissue, which was consistent with the results of TIMER (Fig. 6A–F). The expression levels of COX-2 and GLUT10 were strongly correlated in LUAD samples and their adjunct tissues (r = 0.9197, P < 0.0001) (Fig. 6I). In colocalization analysis, the average Pearson’s correlation coefficient of COX-2 and GLUT10 was 0.960 for the LUAD samples and 0.983 for the adjunct tissues, while the average Manders overlap coefficient was 0.970 for the LUAD samples(p = 0.0093) and 0.985 for the adjunct tissues (P = 0.0331). (Fig. 6G and H, 6J-M).

## 4 Discussion

GLUT10 is encoded by the SLC2A10 gene and is a main transporter of glucose and DHA. Mutations in SLC2A10 cause ATS [20]. SLC2A10 is expressed in various tissues, such as the brain, lung, adipose tissue, heart, placenta and VSMCs [21,22]. Gamberucci et al. [23] found that GLUT10 is mainly expressed in the endoplasmic reticulum. Previous studies have confirmed that GLUT10 is involved in oxidative stress, vitamin C transportation, extracellular matrix degradation and TNF-β signal transduction [24,25]. Syu et al. [26] found that missense mutation of SLC2A10 increases reactive oxygen species production, mitochondrial fragmentation, cell proliferation, and cell migration. Recent research also discovered that SLC2A10 expression levels may be a useful prognostic biomarker in acute myeloid leukemia [27]. It has been found that the characteristics of some cancers are correlated with the expression levels of glucose transporters. For example, GLUT1 and GLUT3 increase the aggressiveness and invasiveness of tumors, thus predicting worse prognosis in patients [28]. However, GLUT10 acts as a member of the glucose transporter family, and few studies have focused on the relationship between SLC2A10 expression and tumor immunity.

Recently, we found that GLUT10 may be a new immune regulator by transcriptome sequencing and bioinformatics studies. We found that SLC2A10 expression in carcinoma samples was significantly different from that in adjacent tissues. The expression level of SLC2A10 is closely correlated with cancer prognosis. Remarkably, we found that regardless of what type of survival (OS, PPS, FP) was considered, the prognosis of lung cancer was significantly related to the expression level of SLC2A10. Compared with LUSC, lower expression of SLC2A10 is more significantly correlated with poor prognosis of LUAD.

The tumor microenvironment (TME) significantly affects the treatment response and prognosis of cancers. In addition to tumor cells, the TME is also composed of immune cells, fibroblasts, endothelial cells, a large number of cytokines, chemokines, and growth factors [29]. As part of the TME, immune cells have a significant impact on cancer prognosis. Based on the relationship analysis between SLC2A10 and immune cell infiltration, we found that the infiltration degree of most immune cells with high SLC2A10 expression was significantly higher, such as activated CD4^+^ T cells, macrophages, neutrophils and DCs. Macrophage infiltration and polarization are mostly significantly influenced by SLC2A10 expression. These findings further convince us that GLUT10 plays an important role in the immune response. To date, few studies have reported the immunomodulatory function of GLUT10. Our findings may add some new knowledge to tumor immunology.

Immunotherapy is a powerful treatment for several cancers, especially LUAD. At present, the common immune checkpoints include PD1/PDL-1, CTLA-4, LAG-3, IM-3 and TIGIT [30].

To further investigate the relationship between SLC2A10 and lung cancer immunity, we used TIMER and GEPIA to explore the correlation between gene expression and immune marker sets of various immune cells of LUAD and LUSC. It is interesting that SLC2A10 expression is significantly correlated with macrophage polarization in cancers. Macrophages are the most characteristic tumor-infiltrating immune cells and the main component of immune cell infiltration in the TME. It plays a significant and positive role in early carcinogenesis tumor progression and metastasis [31]. In the process of tumor progression, circulating monocytes and macrophages are actively recruited into the tumor, changing the TME and accelerating tumor progression. Macrophages alter their functional phenotypes in response to various microenvironmental signals produced by tumor and stromal cells [32]. Macrophages are divided into classically activated (M1) macrophages and selectively activated (M2) macrophages. The function of TAMs is very similar to that of M2 macrophages. Clinicopathological studies have shown that TAM accumulation in tumors is associated with poor clinical outcomes [33]. Under the control of the TME, macrophages dynamically polarize between the M1 phenotype and the M2 phenotype. Xu et al. [34] reported that M2 macrophages promoted the invasion of lung cancer cells and tumor growth, and M1 macrophages inhibited the proliferation and cell activity of lung cancer cells in vivo and in vitro, reduced angiogenesis, increased the chemical sensitivity of lung cancer cells, and induced the apoptosis and senescence of lung cancer cells. Notably, we found that GLUT10 expression is strongly correlated with M1 macrophages but not M2 macrophages in lung cancer. A correlation study using GEPIA also showed that M2 macrophages have a strong correlation with SLC2A10 expression in normal tissue but not in LUAD. In contrast, M1 macrophages were correlated with SLC2A10 expression in LUAD tissue but not in LUSC tissue. Therefore, we speculated that the high expression of SLC2A10 might promote the transformation of M2 macrophages into M1 macrophages. COX-2, an inducible enzyme encoded by the PTGS2 gene, can be highly induced by proinflammatory cytokines, tumor promoters, mitogens, and growth factors in various cells and thus participates in various pathological processes, such as the inflammatory response, cell proliferation and cell apoptosis [35]. Numerous studies indicate that COX-2 is highly expressed in a variety of human cancers, including colorectal, breast, prostate and lung cancer [36,37]. Interestingly, we found that the SLC2A10 expression level is correlated with COX-2 signaling activation, and it is possible that GLUT10 modulates immune infiltration via the COX-2 pathway.

To overcome the limitations of database research, we further verified our findings by analysing the sequencing data of 53 groups of LUAD and the respective adjacent noncancerous tissue in a tissue microarray. We confirmed that the expression of COX-2 and GLUT10 in LUAD was significantly higher than that in adjacent noncancerous tissues. Interestingly, a positive correlation and significant colocalization between GLUT10 and COX-2 expression were also confirmed by our sample research. As an immune marker of M1 macrophages, COX-2 is involved in immune regulation in lung cancer and plays an important role in the early stage of lung cancer. It is possible that GLUT10 modulates the immune cell infiltration of LUAD via the COX-2 pathway.

**Fig. 7:**
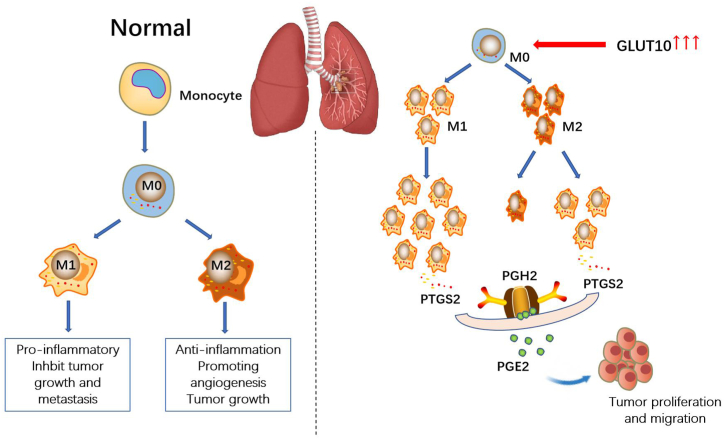
Schematic diagram of the potential mechanisms of GLUT10-mediated promotion of tumorigenesis in lung cancer.

Here, we systematically analysed GLUT10 expression and its relationship with prognosis and immune cell infiltration and preliminarily explored the effect and mechanism of GLUT10 in tumors (Fig. 7). However, our study also has some limitations. The main limitation is that this is database research and there is a lack of arterial model verification. The second limitation is the lack of research on the relationship between Glut10 expression and the effectiveness of PD-1 therapy. This is mainly due to the lack of relevant data in the database at present.

## 5 Conclusion

By transcriptome experiments, database studies, and human sample studies, we found that GLUT10 is a new immune signaling molecule involved in tumor immunity, especially in the immune cell infiltration of LUAD. GLUT10 may modulate the immune cell infiltration of LUAD via the COX-2 pathway.

## Ethics approval and consent to participate

The experimental protocols were approved by the Ethics Committee of Renmin Hospital of Wuhan University.

## Declarations

### Author contribution statement

Lijuan Jian: Performed the experiments; Wrote the paper.

Qi Wu: Performed the experiments; Contributed reagents, materials, analysis tools or data.

Xinping Min, Bowen Li: Contributed reagents, materials, analysis tools or data.

Min Zhang, Zhiwei Wang: Performed the experiments.

Zhiyong Wu, Xiaoping Hu, Zongli Ren: Analysed and interpreted the data.

Zhipeng Hu: Conceived and designed the experiments; Wrote the paper.

### Funding statement

This work was supported by National Natural Science Foundation [82100423 & 81600367].

### Data availability statement

Data will be made available on request.

### Declaration of interest’s statement

The authors declare no competing interests.
